# Honorary Medical Staffs of Hospitals

**Published:** 1920-06-05

**Authors:** 


### Honorary Medical Staffs of Hospitals.

THE QUESTION OF PAYMENT.

For several years the payment of Honorary Medical and Surgical Staffs of Voluntary Hospitals has received a good deal of attention. At a conference recently held at the Leicester Royal Infirmary, attended by representatives of the Honorary Staffs of the larger Voluntary Hospitals of the Midlands, the question was fully debated and considered.

Representatives attended from hospitals at Birmingham, Burton-on-Trent, Coventry, Derby, Leicester, Loughborough, Manchester, Northampton, Nottingham, Salford, Sheffield, Stoke-on-Trent, West Bromwich, Wolverhampton.

Sir Richard Luce, K.C.B., of Derby, who was in the chair, brought forward the following points for discussion :

**Honorary Medical Staffs of Hospitals**—(*continued*).

The desirability of payment for the Visiting Staffs for work done at their Hospitals *on behalf of* the Ministry of Pensions, the Ministry of Health, or the local Medical Health Authorities, and at what rates.

*Whether* the time has come to ask for payment for visiting staffs for *all work* done by them at their Hospitals; and, if so, whether this should take the form of an honorarium, a salary, or a percentage of the expenses of the Hospital. Alternatively, what alteration in the existing management or financial arrangements of the Hospital would create the need for a request for such payment.

The matter was very fully debated and discussed, and the following resolutions were adopted :

1. "That Payment for Visiting Staffs was desirable for work done at their Hospitals on behalf of the Ministry of Pensions, the Ministry of Health, or the Local Medical Health Authorities. Methods of payment and rates of pay were referred to a Committee for detailed consideration and report."

2. "That the time has not yet come for visiting staffs to ask for payment for all work done by them at the Hospitals, but that the need for a request for payment would arise if the State or Local Authorities should assume control over the Hospitals or if the income of the Hospitals ceased to be derived from voluntary contributions."

The question of a permanent organisation representing the interests of Honorary Medical Staffs of Hospitals was considered, and decided in the negative, but the Chairman and Secretary were authorised to reassemble the conference at any time at their discretion.

